# Supplementary material for: The Construction and Exploration of a Comprehensive MicroRNA Centered Regulatory Network in Foxtail Millet (Setaria italica L.)
Source: Front Plant Sci. 2022 May 6;13:848474. doi: 10.3389/fpls.2022.848474 (PMC9121102; doi:10.3389/fpls.2022.848474)
Supplement: Supplementary file 1 [file Data_Sheet_1.zip › Sit-miR396d_Seita.1G271800.1_648_TPlot.pdf]

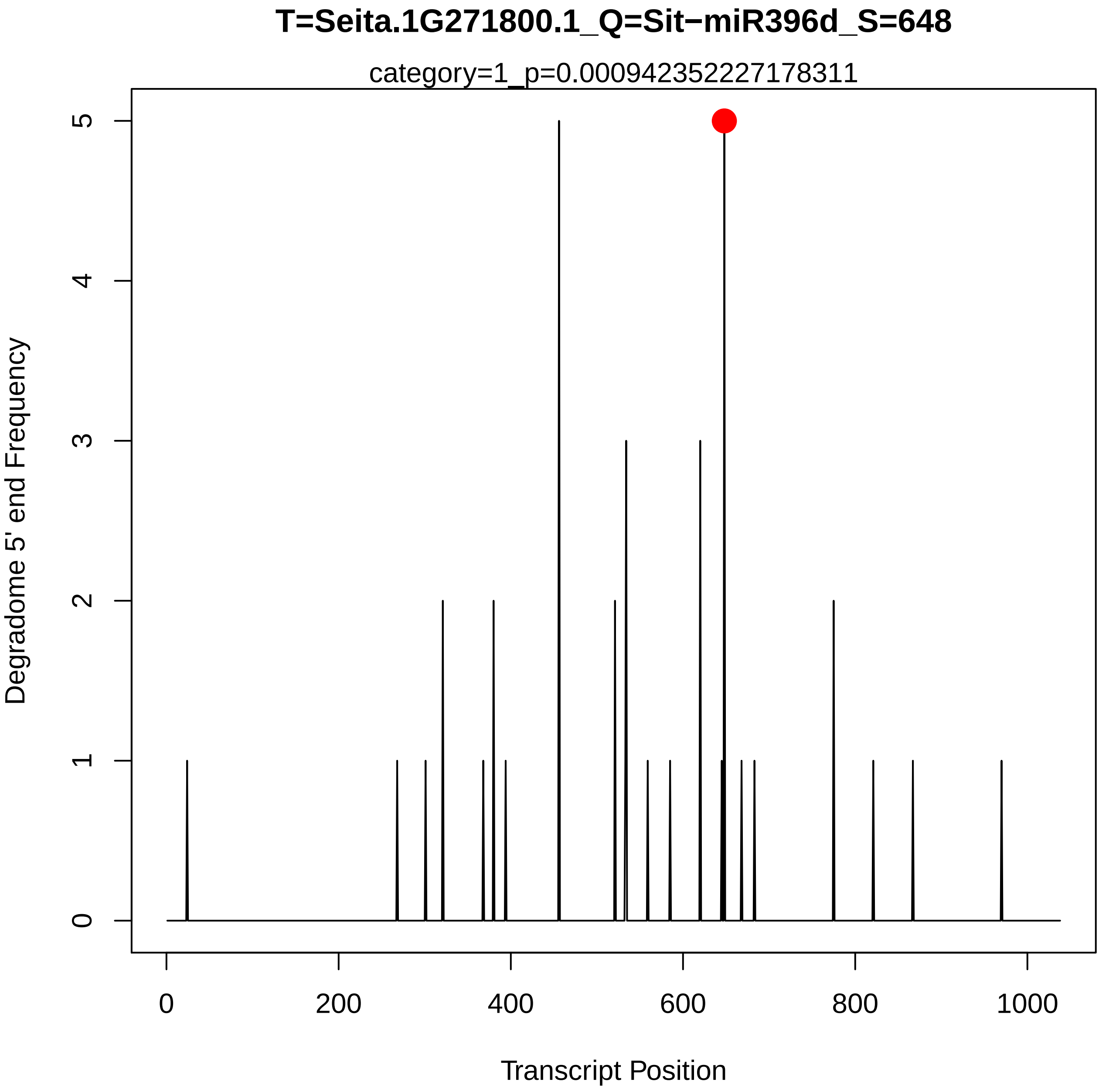

T=Seita.1G271800.1_Q=Sit−miR396d_S=648
category=1_p=0.000942352227178311
Degradome 5' end Frequency
Transcript Position
0
1
2
3
4
5
0
200
400
600
800
1000
